# Nutritional Avocado Intervention Improves Physical Activity Measures in Hispanic/Latino Families: A Cluster RCT

**DOI:** 10.1016/j.focus.2023.100145

**Published:** 2023-09-20

**Authors:** Tara Shrout Allen, Aubrey L. Doede, Colin M.B. King, Lorena S. Pacheco, Gregory A. Talavera, Julie O. Denenberg, Amelia S. Eastman, Michael H. Criqui, Matthew A. Allison

**Affiliations:** 1 Division of Preventive Medicine, Department of Family Medicine, University of California, San Diego, La Jolla, California; 2 School of Public Health, University of California, San Diego, La Jolla, California; 3 Department of Nutrition, Harvard T.H. Chan School of Public Health, Boston, Massachusetts; 4 Department of Psychology, San Diego State University, San Diego, California; 5 Department of Family Medicine, University of California, San Diego, La Jolla, California; 6 Division of Cardiovascular Medicine, Department of Medicine, University of California, San Diego, La Jolla, California

**Keywords:** Physical activity, sedentary behavior, nutrition, lifestyle intervention

## Abstract

**Introduction:**

Nutrition and physical activity are key components for the prevention of cardiovascular disease. There remains a paucity of trial data on the effect of specific nutritional interventions on physical activity and sedentary time. One question is how a common nutrient-dense food such as avocado may impact physical activity and sedentary time in Hispanic/Latino families, a group that reports the lowest levels of physical activity.

**Design:**

This is a 6-month clustered RCT.

**Setting/participants:**

Seventy-two families (235 individuals) who identified as Hispanic/Latino were enrolled through the San Ysidro Health Center (San Diego, CA) between April 2017 and June 2018.

**Intervention:**

After a 2-week run-in period, 35 families were randomized to the intervention arm (14 avocados/family/week), and 37 families were assigned to the control arm (3 avocados/family/week).

**Main outcome measures:**

Linear mixed-effects models were used to assess changes in physical activity (MET minutes per week) between the groups during the 6-month trial*.* Secondary outcomes included sedentary time (minutes/week), BMI, and systolic and diastolic blood pressures.

**Results:**

An adherence goal of >80% was achieved for both arms. Total mean physical activity increased by 2,197 MET minutes per week more in the intervention group (*p*<0.01) than in the control group, driven by between-group differences in moderate (*p*<0.01) versus vigorous (*p*=0.06) physical activity. After accounting for longitudinal repeated measures per participant and nested family effects, total adult physical activity remained significantly higher in the intervention than in the control group (+1,163 MET minutes per week on average per participant), with a significant intervention interaction term (*p*<0.01). There were no significant changes in sedentary time, BMI, or blood pressure.

**Conclusions:**

Higher allocation of avocados was associated with significantly higher physical activity and no adverse changes in BMI or blood pressure, suggesting that this nutritional intervention may have beneficial pleiotropic effects.

**Trial registration:** This study is registered at www.clinicaltrials.gov as NCT02903433.

## HIGHLIGHTS

• Higher avocado intake led to significantly increased physical activity levels.
• Hispanic/Latino adults may benefit from higher avocado intake.
• More data are needed on the relationship between avocados, physical activity, and health effects in Hispanic/Latino families.

## Graphical Abstract

**Figure:**
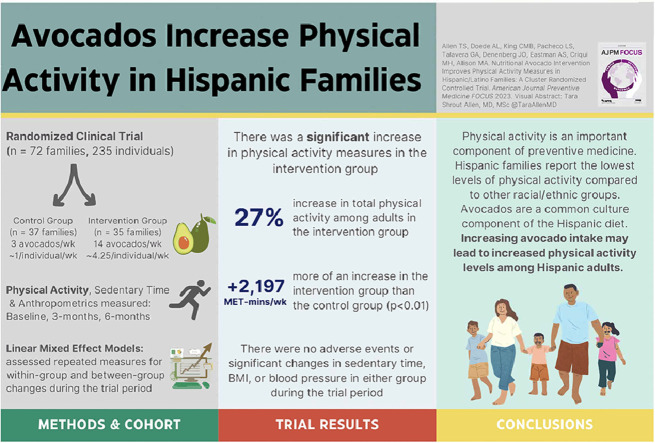

## INTRODUCTION

Physical activity (PA) is an established method to prevent adverse cardiovascular outcomes and chronic disease.^1^, ^2^, ^3^, ^4^, ^5^, ^6^ Moreover, insufficient PA accounts for approximately $117 billion in annual healthcare costs and 10% of premature mortality in the U.S.^7^, ^8^, ^9^ Notably, U.S. Hispanic/Latino populations report the highest prevalence of physical inactivity at 31.7%, compared with 23.4% in non-Hispanic White populations.^10^ In fact, only 21.3% of Hispanic adults report achieving the recommended PA levels.^11^ As the largest U.S. minority ethnic group^12^^,^^13^ and generally at higher cardiometabolic disease risk,^14^^,^^15^ it is an important public health objective to assess modifiable lifestyle interventions in these populations that may improve PA outcomes and prevent the burden of chronic disease.

Higher PA levels are associated with improved nutritional intake in both children and adults.^16^^,^^17^ However, there is a paucity of longitudinal trial data on whether specific nutritional interventions may lead to favorable PA outcomes, especially among Hispanic/Latino individuals. One nutrient-dense food that is common to these groups is avocado. Data suggest that >90% of Hispanic/Latino households purchase avocados on a regular basis,^18^ with an average intake of 3 avocados per week.^19^ Data also suggest that regular avocado intake—even one half of an avocado daily^20^^,^^21^—supports cardiovascular health, including improved lipid profiles,^20^, ^21^, ^22^, ^23^, ^24^ enhanced endothelial function,^21^^,^^25^ and lower metabolic syndrome risk.^21^^,^^26^^,^^27^ As such, a nutritional lifestyle intervention related to avocados is applicable in Hispanic/Latino populations and may result in beneficial pleiotropic outcomes.^28^

This study aimed to assess how the provision of 2 different levels of avocados among Latino families affected specific PA outcomes through a cluster RCT. Secondary outcomes, including sedentary time, BMI, and blood pressure measurements, were also assessed.

## METHODS

### Study Population

This study was a cluster RCT conducted in San Diego County, California, entitled “The Effects of Avocado Intake on the Nutritional Status of Families Trial.” The original objective of this trial was to assess the changes in the comprehensive nutritional status between the groups.^19^ The randomization unit was the family, and the intervention was the number of avocados allotted to each family per week (i.e., 14 vs 3). Participants were followed for a total of 6 months with clinic visits at baseline (Visit 1), 3 months (Visit 2), and 6 months (Visit 3). Throughout the trial, adherence data were obtained with allotment deliveries.

All protocols and materials were approved by the IRBs of the University of California, San Diego and San Diego State University. The clinical trial was registered at www.clinicaltrials.gov as NCT02903433 on September 16, 2016, with the first participant enrolled on April 20, 2017 (https://www.clinicaltrials.gov/ct2/show/NCT02903433).

**Figure 1:**
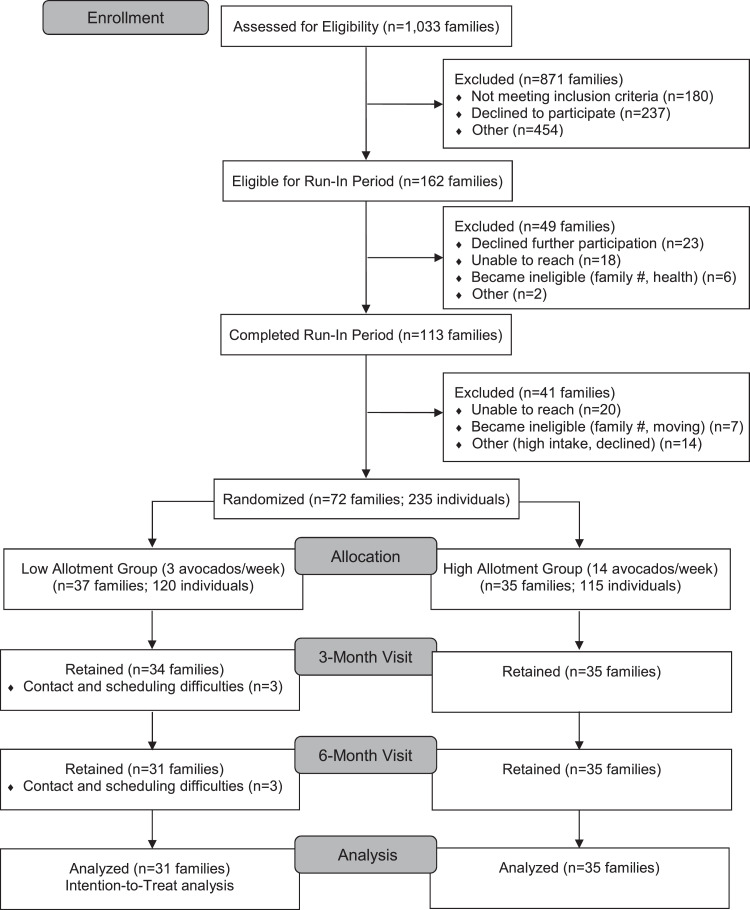
CONSORT flow diagram.

Participants were recruited through the San Ysidro Health Center, San Diego, California, between April 2017 and June 2018^19^ (Figure 1). Inclusion criteria were as follows: families who self-identified as Hispanic/Latino with 3–8 individual members living in the same household, all aged ≥5 years, and all willing to participate in the intervention. Exclusion criteria were as follows: allergy to avocado or latex, current high avocado consumption (>1 avocado per individual per day for adults or more than half of an avocado per day for children so as to not result in decreased intake from baseline), specific health factors (presence of family member with severe chronic disease requiring specific diet or presence of family member who is pregnant or intending to become pregnant), or a plan to move within the 6-month trial period.

Before the start of the trial, a 2-week run-in period was completed, which assessed adherence to study procedures, including dietary intake of allocated avocados. Adherent families who completed the run-in period were scheduled for the baseline visit for enrollment and to obtain general demographic information as well as baseline clinical measurements. The *head of household* (HOH)—defined as the family member who primarily shops for household groceries and prepares family meals—was identified during enrollment, and additional HOH demographic data were obtained. *Age groups in the cohort* were defined as the following: adults (aged ≥18 years), adolescents (aged 13–17 years), and children (aged 5–12 years). Each participant consented or assented appropriately to the study.

Family randomization occurred at the baseline visit using SAS programming with a blocked, randomization sequence through the RANUNI function, as previously described.^19^ Randomization assignments were blinded to staff members and study researchers. Study arm assignment was applied by the study coordinator. Community health workers and participants were unmasked to the intervention assignment owing to necessary delivery of specific avocado allotments and adherence instructions. Each of the trial visits was carried out by blinded study personnel not involved in intervention assignment or implementation.

The number of avocados in the control group was determined on the basis of an intake survey (*n*=101), which revealed an average avocado intake of 3 per week per family.^19^ As noted, the intervention group was allotted 14 avocados per family per week on the basis of a robust increase from baseline and expected mean family size to derive allotted intake of approximately half to three fourths of an avocado per individual per day. Both intervention groups were encouraged to limit the intake of additional avocados or avocado-based products during the trial to reduce the risk of variability and group contamination. Adherence to study arm assignment was assessed throughout the trial. A group without avocado allotment was considered, although it was deemed not reasonable given the reported typical intake by the target population.

All families also received a standardized home-based nutritional education program over the 6-month trial period.^19^ The concurrent nutritional education intervention aimed to prevent unintentional adverse effects of dietary avocado incorporation, such as an increased intake of chips with avocado or guacamole, which could lead to increased sodium intake and potentially adverse clinical secondary outcomes. Briefly, the program consisted of 12 in-home sessions covering culturally appropriate nutrition information with materials provided from the U.S. Department of Agriculture MyPlate platform,^29^ and each session was delivered by a trained community health worker who also home delivered the avocado allotments.^30^ In addition, families received a recipe booklet with ideas on how to incorporate avocados into their diet and a care guide to assist with proper avocado maturation.

Study retention strategies included the following: communication with staff through telephone, e-mail, and in-person trial visits with language preference of English or Spanish per family preference; provision of avocado allotments per intervention assignment delivered by community health workers; ongoing nutritional education sessions; and a monetary incentive of $100 provided at the 3-month (Visit 2) and 6-month (Visit 3) time points.

### Measures

The primary outcome measure for this analysis was PA, which was assessed among all participants and measured as MET minutes per week using the data obtained from the validated Global Physical Activity Questionnaire (GPAQ).^31^, ^32^, ^33^ All participants in the trial individually completed the GPAQ during the baseline, 3-month, and 6-month clinic visits, with PA data captured as minutes per day. Total PA as well as specific component measures of occupational, recreational, and transportation-related PA were assessed. These measures were further categorized as moderate (associated with 4 METs) or vigorous (associated with 8 METs). Longitudinal MET minutes per week were then calculated for each PA measure category for each individual participant. All participants completed information on sedentary time at each clinic visit also through the GPAQ, captured as minutes per day spent in a sitting or reclining sedentary position, for which a measure of minutes per week was calculated.

Ongoing avocado adherence was measured using 2 separate tools.^19^ Individual avocado intake per week was assessed during trial visits using the validated VioScreen Food Frequency Questionnaire. As a second measure, an Avocado Daily Diary (Appendix Figure 1, available online) assessed avocado adherence at the family unit on the basis of the number of avocados delivered, consumed, and remaining unconsumed, as previously described.^19^ Data from the Avocado Daily Diary were collected from HOHs every 2 weeks, obtained during the weekly home allotment delivery by a community health worker and then provided to the blinded research team. A continuous adherence value was calculated on the basis of the amount consumed by each family divided by their study arm intake goal. The VioScreen Food Frequency Questionnaire additionally captured the estimated total daily intake of energy in kilocalories at the individual level at baseline, 3-month, and 6-month trial endpoint.

At the baseline visit, each HOH provided information on family sociodemographic factors, family dietary and lifestyle behaviors, and HOH-specific demographics. During each of the trial visits, systolic blood pressure (SBP) and diastolic blood pressure (DBP) were measured (in millimeters of mercury [mmHg]) 3 times at rest. Furthermore, during each trial visit, BMI was calculated from the measures of weight (lb) and height (in).

### Statistical Analysis

Descriptive statistics were used to characterize the sample by study arm assignment. The chi-square test was used to compare proportions for categorical variables. Normality was evaluated for all continuous variables through the Shapiro–Wilk method. Given the number of observations, the 2-sample *t*-test was used to assess mean differences in continuous variables. Categorical variables were expressed as frequencies and percentages, whereas continuous variables were expressed as means and SDs.

Primary outcome analyses were conducted using longitudinal data with an intention-to-treat approach. First, group mean differences between baseline and follow-up PA measures at 3 months and 6 months were assessed, comparing the intervention groups using 2-sample *t*-tests. Mean differences and SDs are presented. Second, to appropriately assess longitudinal repeated-measures ANOVA was used to compare linear mixed-effects (LME) models with and without allowing intercepts to vary by family and confirm the need for a multilevel model to account for the effect of clustered families given this introduced dependency. Generalized LME models were used to assess primary and secondary outcomes. This study controlled for the nested effect of both family and individual participants and accounted for all covariates that were statistically significant in the univariate models. We assessed results among age stratification and complete cases. A separate sensitivity analysis additionally adjusted for total daily energy intake in kilocalories (accounting for longitudinal data from baseline, 3 months, and 6 months) for the primary outcomes of total PA among all participants and then just among adults. All *p*-values <0.05 were considered statistically significant. Analyses were performed using R. A 2-tailed *p*<0.05 was considered statistically significant.

## RESULTS

The trial enrolled 72 families (235 participants) between April 11, 2017, and June 27, 2018, with 37 families randomized to the control group (120 participants) and 35 families randomized to the intervention group (115 participants) (Figure 1). At baseline, there were no statistically significant differences in demographics, anthropometric measures, PA levels, or sedentary time between the trial groups (Table 1).

**Table 1:** Baseline Characteristics of Trial Participants Per Study Group

Characteristics	Control group (*n*=37 families; 120 participants)	Intervention group (*n*=35 families; 115 participants)	*p*-value^a^
	*n* (%) or mean (SD)		
Female sex	70 (58.3%)	80 (69.6%)	0.07
Family income <$30,000/year	15 (12.5%)	19 (16.5%)	0.60
Participant age groups			0.56
Adults^b^	74 (61.7%)	67 (58.3%)	
Adolescents	14 (11.7%)	19 (16.5%)	
Children	32 (26.7%)	29 (25.2%)	
Mean family size	3.2±0.6	3.3±0.6	0.77
Age, years			
All participants	32.3±21.0	29.1±18.3	0.22
Adults	45.4±15.9	41.4±14.3	0.15
Adolescents	16.0±1.0	15.5±1.4	0.48
Children	8.9±2.1	9.5±2.2	0.36
Adult BMI, kg/m^2^	31.0±6.1	29.9±6.6	0.18
Waist-to-height ratio, cm			
Adolescents	0.50±0.12	0.49±0.07	0.90
Children	0.48±0.06	0.49±0.09	0.55
Systolic BP, mmHg			
Adults	120.0±16.1	116.7±19.2	0.28
Adolescents	108.7±7.9	109.4±8.4	0.82
Children	98.8±7.6	101.5±7.9	0.19
Diastolic BP, mmHg			
Adults	72.7±11.3	71.2±9.2	0.40
Adolescents	62.7±9.5	65.7±6.5	0.29
Children	58.2±7.7	61.7±9.6	0.12
Adult PA, MET minutes/week			
Total activity	5,460±6,963	4,114±5,344	0.20
Occupational, moderate	1,859±3,052	1,364±2,416	0.29
Occupational, vigorous	2,052±5,295	1,048±2,832	0.16
Recreational, moderate	356±547	367±608	0.91
Recreational, vigorous	822±1,721	716±1,252	0.68
Transportation-related	411±722	639±1,553	0.28
Adolescent PA, MET minutes/week			
Total activity	4,099±4,022	3,147±2,818	0.68
Recreational, moderate	868±1,312	491±956	0.42
Recreational, vigorous	1,828±2,203	1,448±1,827	0.55
Transportation-related	412±692	640±1,263	0.52
Occupational, moderate^c^	330±467	336±602	0.98
Occupational, vigorous^c^	160±426	232±619	0.71
Child PA, MET minutes/week			
Total activity	2,541±3,107	1,869±2,510	0.40
Recreational, moderate	558±893	601±1,338	0.89
Recreational, vigorous	1,266±2,137	1,060±1,147	0.65
Transportation-related	725±1,359	288±716	0.13
Sedentary time, minutes/week			
All participants	1,870±1,138	1,976±1,208	0.50
Adults	1,487±939	1,857±1,275	0.06
Adolescents	2,415±1,227	2,147±1,136	0.53
Children	2,541±1,153	2,145±1,092	0.18

a From 2-sample *t*-test or chi-square test where appropriate.

b Adult participants: control group, *n*=74; intervention group, *n*=67.

c Occupational activity was recorded for adolescent participants (age range=13–17 years). BP, blood pressure; PA, physical activity.

On average, families comprised 3.2 individuals (SD=0.6; range=3–6), with a mean age of 30.7 years. Sixty percent of household members were categorized as adults (mean age=43.5 years; SD=15.2; range=18–88 years), 14% were categorized as adolescents (mean age=15.8 years; SD=1.2; range=13–17 years), and 26% were categorized as children (mean age=9.2 years; SD=2.2; range=5–12 years). Female sex trended toward a higher proportion in the intervention arm (*p*=0.07). Family income was <$30,000 in 34 families and ≥$30,000 in 28 families, for which there was no significant difference between groups; 10 families did not provide income information. On average, adults were slightly obese (BMI=30.2 kg/m^2^; SD=6.3; range=19.4–48.4 kg/m^2^), whereas children and adolescents had a modestly elevated waist-to-height ratio of 0.49 cm (SD=0.07) and 0.50 cm (SD=0.10), respectively.^34^^,^^35^ Overall, participants were normotensive (SBP=112.6 mmHg [SD=16.6] and DBP=67.8 mmHg [SD=11.0]), with no significant differences between groups (Table 1).

The total mean PA at baseline among adult participants was 4,816 MET minutes per week (SD=6,255), with a median of 2,490 MET minutes per week—values that meet both current American Heart Association and WHO recommendations for adult populations.^35^^,^^36^ Among the specific categories comprising total PA, occupational activity contributed the most, with a mean of 3,171 MET minutes per week (SD=5,712), followed by recreational activity with 1,133 MET minutes per week (SD=1779) and then transportation-related activity with 521 MET minutes per week (SD=1201) (Table 1). Baseline total mean PA was lower among adolescents (3,354±3,211 MET minutes per week) and children (2,248±2,856 MET minutes per week) than among adults. Sedentary time at baseline among all participants was on average 1,921 minutes per week (SD=1,171), with a median of 1,680 minutes per week.

### Study Retention

Of the 72 enrolled families, 69 (95.8%) attended the 3-month visit, and 66 (91.7%) attended the 6-month visit (Figure 1). Family attrition was 16.2% in the control group and 0% in the intervention group at 6 months (*p*=0.03). Families and HOHs who dropped out were not significantly different from those who remained by baseline demographics, anthropometrics, sedentary time, or total PA among adults and children; however, baseline total PA among adolescents was significantly different between the groups (*p*=0.04) (Appendix Table 1, available online). Reported reasons for dropout included time constraints, scheduling conflicts, and difficulty contacting families. No harm or unintended effects were reported by study participants.

### Adherence Analyses

Individual avocado intake per week was reported throughout the trial in all participants. Adherence was met by 95% of the control group families and 83% of the intervention group families at the 6-month visit, both of which were greater than the study goal of 80%. Moreover, 61 families (85%) had ≥80% continuous adherence to the intervention protocol throughout the duration of the study, corresponding to 92% and 77% for the control and intervention families, respectively. Individuals in the intervention group had an average intake of 4.10 and 4.03 avocados per week at the second (3-month) and third (6-month) clinic visits, respectively, which was slightly below the goal of approximately 4.24 avocados per week per individual accounting for mean family size. In contrast, individuals in the control group had an average intake of 1.25 and 1.48 avocados per week at the 3- and 6-month clinic visits, respectively, which was slightly above the goal of approximately 0.94 avocados per week per individual (Appendix Figure 2, available online).

### Mean Differences in Adult Physical Activity Measures Between Study Groups

**Figure 2:**
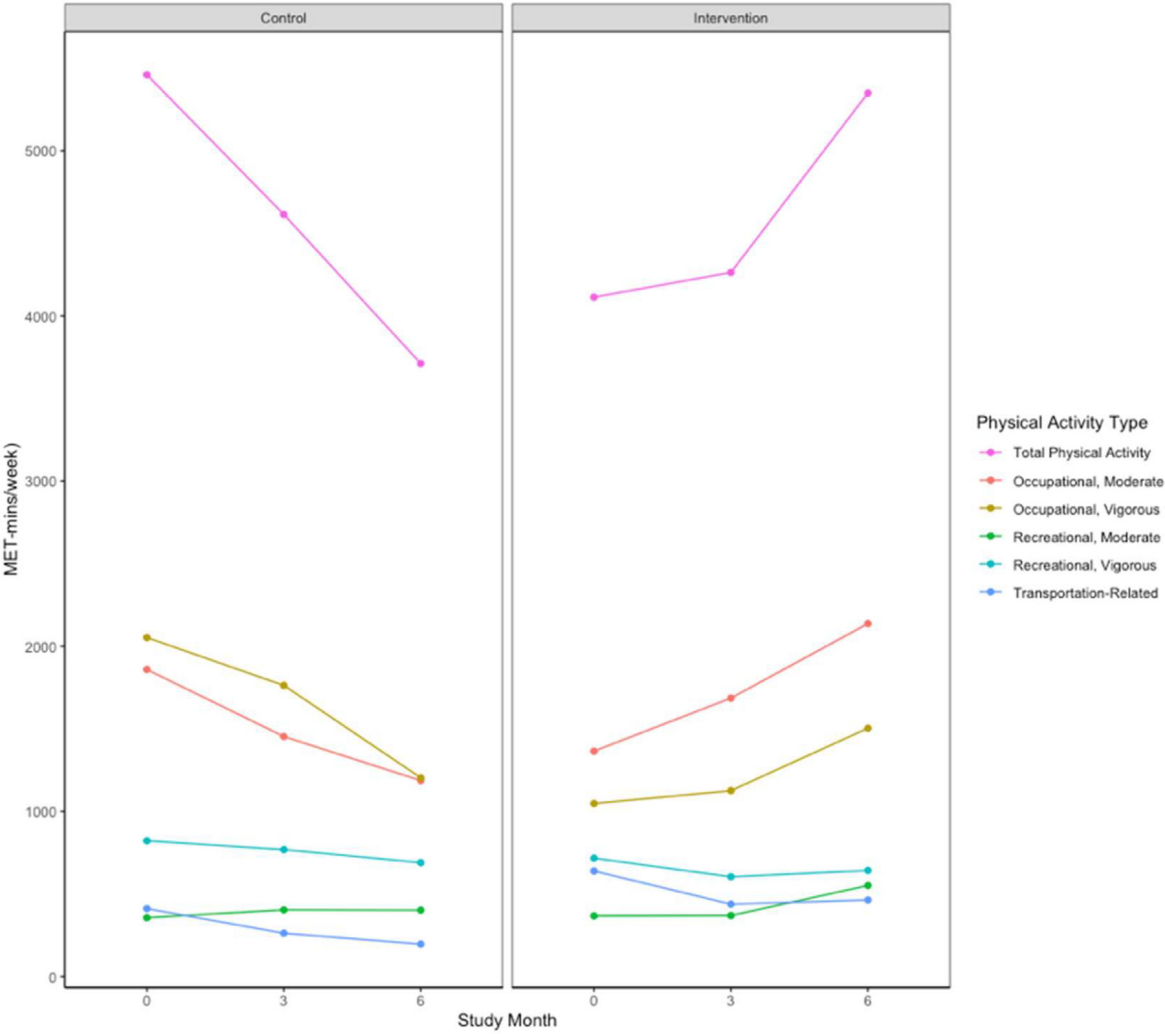
Trends in PA measures among adults per study arm assignment. *Note*: Whereas total PA decreased in the control group, it increased in the intervention group. Component PA types that contributed to total PA are shown. PA units are provided as MET-min/wk. Whereas mean total PA significantly decreased in the control group, it significantly increased in the intervention group. Component PA types that contributed to total PA are shown. By the end of the trial, mean total PA improved by +2,197 MET-min/wk more in the intervention group than in the control group (*p*<0.001), which was driven by changes between groups in moderate PA (*p*<0.001) rather than in vigorous PA (*p*=0.056). 3mo, 3 months; 6mo, 6 months; MET-mins/wk, MET minutes per week; PA, physical activity.

Although mean PA significantly increased in the intervention group during the trial, the inverse occurred in the control group (Figure 2). Indeed, by the end of the trial, total mean PA improved by 2,197 MET minutes per week more in the intervention than in the control group (*p*<0.001), driven by changes between the groups in moderate PA (*p*<0.001) rather than in vigorous PA (*p*=0.056) (Table 2). Among the specific categories that comprised total PA, significant differences between the study groups were detected in mean values at the trial endpoint for total occupational PA (*p*<0.001), moderate occupational PA (*p*<0.001), vigorous occupational PA (*p*=0.03), and moderate recreational activity (*p*=0.04), each favoring the intervention. There were no significant differences in any PA measures between the groups at the 3-month visit or in transportation-related PA throughout the trial (Table 2).

**Table 2:** Changes in Adult Physical Activity Measures Between Groups

	Within-group differences		Between-group differences *p*-value^a^
Physical activity	Control group (*n*=74 adults)	Intervention group (*n*=67 adults)	
	**Mean ± SD**	**Mean ± SD**	
Total physical activity			
3-month, baseline	−666±6,969	+53.6±5,051	0.24
6-month, baseline	−1,068±4,012	+1,129±6,284	<0.001***
Total moderate activity			
3-month, baseline	−276±3,691	+297±3,086	0.096
6-month, baseline	−487±2,793	+930±3,627	<0.001***
Total vigorous activity			
3-month, baseline	−216±5,192	−63.6±3,801	0.74
6-month, baseline	−333±2,483	+347±4,247	0.056
Occupational, moderate			
3-month, baseline	−306±3,638	+301±3,097	0.08
6-month, baseline	−477±2,538	+752±3,678	<0.001***
Occupational, vigorous			
3-month, baseline	−242±5,031	+60.9±3,479	0.49
6-month, baseline	−248±1,841	+439±4,072	0.03*
Recreational, moderate			
3-month, baseline	+30.5±689	−3.6±730	0.63
6-month, baseline	−9.5±794	+178±962	0.04*
Recreational, vigorous			
3-month, baseline	+22.8±1,532	−124±1,059	0.27
6-month, baseline	−88.8±1,774	−85.5±1,109	0.98
Transportation-related			
3-month, baseline	−179±843	−210±1,682	0.82
6-month, baseline	−284±773	−184±1,929	0.51

*Note:* Boldface indicates statistical significance (*p<0.05, ***p<0.001).

Mean differences between groups are presented as control minus intervention group.

a From unpaired 2-sided *t*-test; ANCOVA models were not necessary because there were no baseline differences.

### Longitudinal Changes in Physical Activity Measures

The effect of clustered families was confirmed significant for total PA (L-ratio=37.20, *p*<0.001). Generalized LME models demonstrated that total PA among all participants significantly improved in the intervention versus control groups (+300 more MET minutes per week on average per participant, *p*=0.006) (Appendix Figure 3, available online) with a significant intervention interaction term (*p*=0.03) (Appendix Table 2, available online). In a sensitivity analysis that additionally adjusted for total calorie intake throughout the study, the effect remained significant when assessed among all participants (*p*=0.02) (Appendix Table 3, available online). The specific PA categories—including moderate PA and moderate occupational PA—when assessed among all participants did not differ between the groups by the trial endpoint (*p*=0.20 and *p*=0.10, respectively) (Appendix Figure 3B and 3C, respectively, available online; Appendix Table 2, available online).

**Figure 3:**
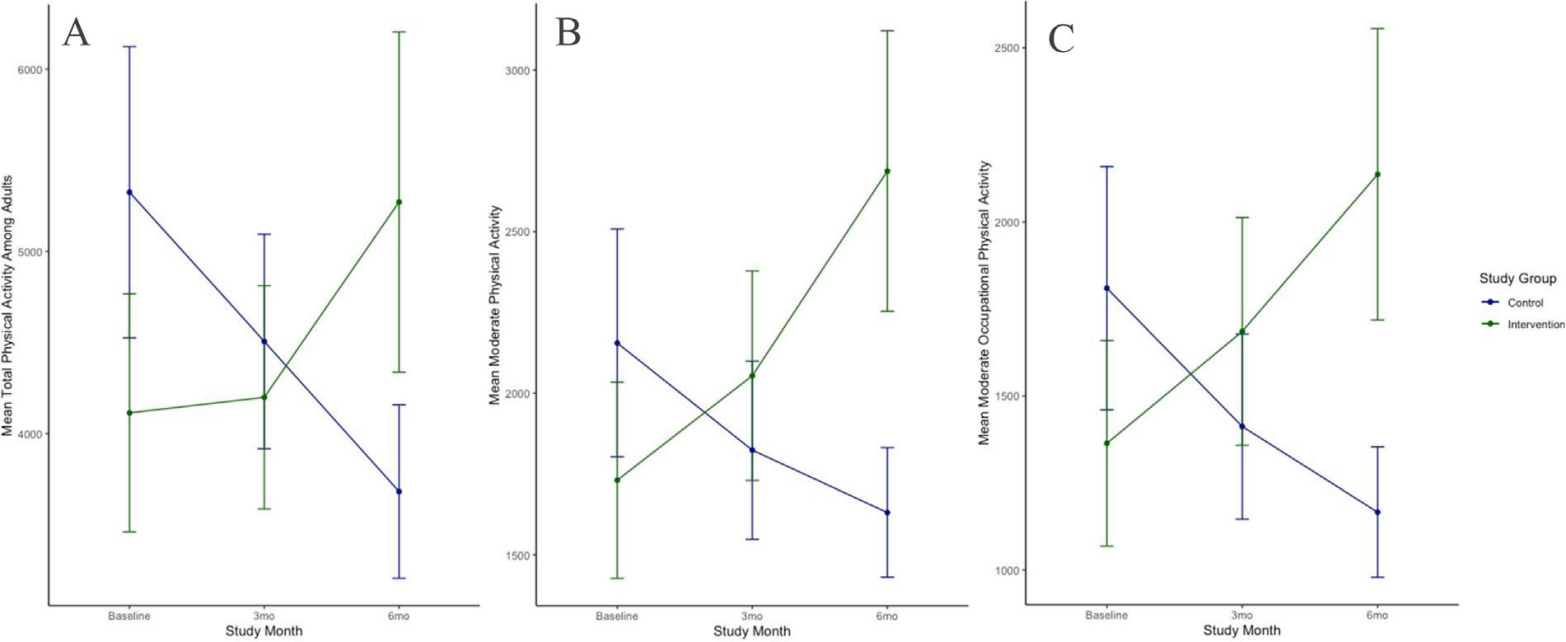
Longitudinal changes in significant adult PA measures. (A) Changes in mean total PA among adults and per study group. (B) Changes in mean total moderate PA among adults and per study group. (C) Changes in mean moderate occupational PA among adults and per study group. PA units are provided as MET-min/wk. *Note*: The key statistical results are as follows: total adult PA significantly improved in the intervention versus the control group (+1,163 MET-min/wk on average per participant) by the trial endpoint and with a significant intervention interaction term (*p*=0.009) (Panel A). Moderate PA (Panel B) and moderate occupational PA (Panel C) also significantly improved in the intervention versus control group (*p*=0.009 and *p*=0.03, respectively). 3mo, 3 months; 6mo, 6 months; MET-mins/wk, MET minutes/week; PA, physical activity.

After stratification by age categories, LME models demonstrated that changes in total PA among adults (*n*=141) remained significantly improved in the intervention versus control groups (+1,163 MET minutes per week on average per participant) by the trial endpoint (*p*=0.03) with a significant intervention interaction term (*p*=0.009) (Table 3, Figure 3A). In the sensitivity analysis that additionally accounted for total calorie intake throughout the study, the effect favoring the intervention group remained significant (*p*=0.03) with a significant intervention interaction term (*p*=0.005) (Appendix Table 3, available online). Among adults, neither age nor BMI modified the associations (interaction terms *p*=0.56 and *p*=0.85, respectively). Among specific types of PA in adults, there was a statistically significant difference between groups for outcomes of moderate PA (Figure 3B) and moderate occupational PA (Figure 3C) (*p*=0.009 and *p*=0.03, respectively). Results for vigorous occupational PA and moderate recreational PA were similar between the groups (*p*=0.12 and *p*=0.30, respectively) (Table 3).

**Table 3:** Longitudinal Changes in Adult Physical Activity Measures

Dependent variable	Coefficient	95% CI	*p*-value
Total PA			
Intervention	−1,340	−3,398, 718	0.20
Study Month 3	−759	−2,155, 637	0.29
Study Month 6	−1,537	−2,991, −83.4	0.03*
Intervention Х study Month 3	877	−1,106, 2,860	0.39
Intervention Х study Month 6	2,700	676, 4,724	**0.009^⁎⁎^**
Moderate PA			
Intervention	−484	−1,407, 439	0.30
Study Month 3	−340	−1,154, 474	0.41
Study Month 6	−613	−1,451, 227	0.15
Intervention Х study Month 3	658	−500, 1815	0.26
Intervention Х study Month 6	1,563	388, 2,738	**0.009^⁎⁎^**
Moderate occupational PA			
Intervention	−542	−1,478, 395	0.25
Study Month 3	−399	−1,269, 471	0.37
Study Month 6	−655	−1,551, 241	0.15
Intervention Х study Month 3	713	−527, 1952	0.26
Intervention Х study Month 6	1,420	163, 1420	0.027*
Vigorous occupational PA			
Intervention	−997	−2,380, 386	0.16
Study Month 3	−267	−1,229, 696	0.59
Study Month 6	−662	−1,658, 335	0.19
Intervention Х study Month 3	335	−1,029, 1699	0.63
Intervention Х study Month 6	1,108	−280, 2,497	0.12
Moderate recreational PA			
Intervention	11.4	−214, 237	0.92
Study Month 3	42.7	−153, 239	0.67
Study Month 6	33.4	−169, 236	0.75
Intervention Х study Month 3	−41.5	−321, 237	0.77
Intervention Х study Month 6	150	−133, 433	0.30

*Note*: Boldface indicates statistical significance (*p<0.05, ***p<0.001).

Summary statistics from linear mixed-effects models for physical activity measures among the control versus intervention group are presented.

PA, physical activity.

LME models further confirmed that total PA among adolescents did not significantly differ between groups at the trial mid-point (*p*=0.22), although it trended toward improvement in the intervention versus control groups by the trial endpoint (*p*=0.08) (Appendix Figure 4B, available online). Similarly, moderate PA among adolescents did not significantly differ between the groups at the trial mid-point (*p*=0.78), although it trended toward improvement in the intervention versus control groups by the trial endpoint (*p*=0.11) (Appendix Figure 4C, available online). Total PA among children did not significantly differ between the groups (*p*=0.39 at the mid-point and *p*=0.55 by the endpoint) (Appendix Figure 4A, available online).

### Longitudinal Changes in Sedentary Time

**Figure 4:**
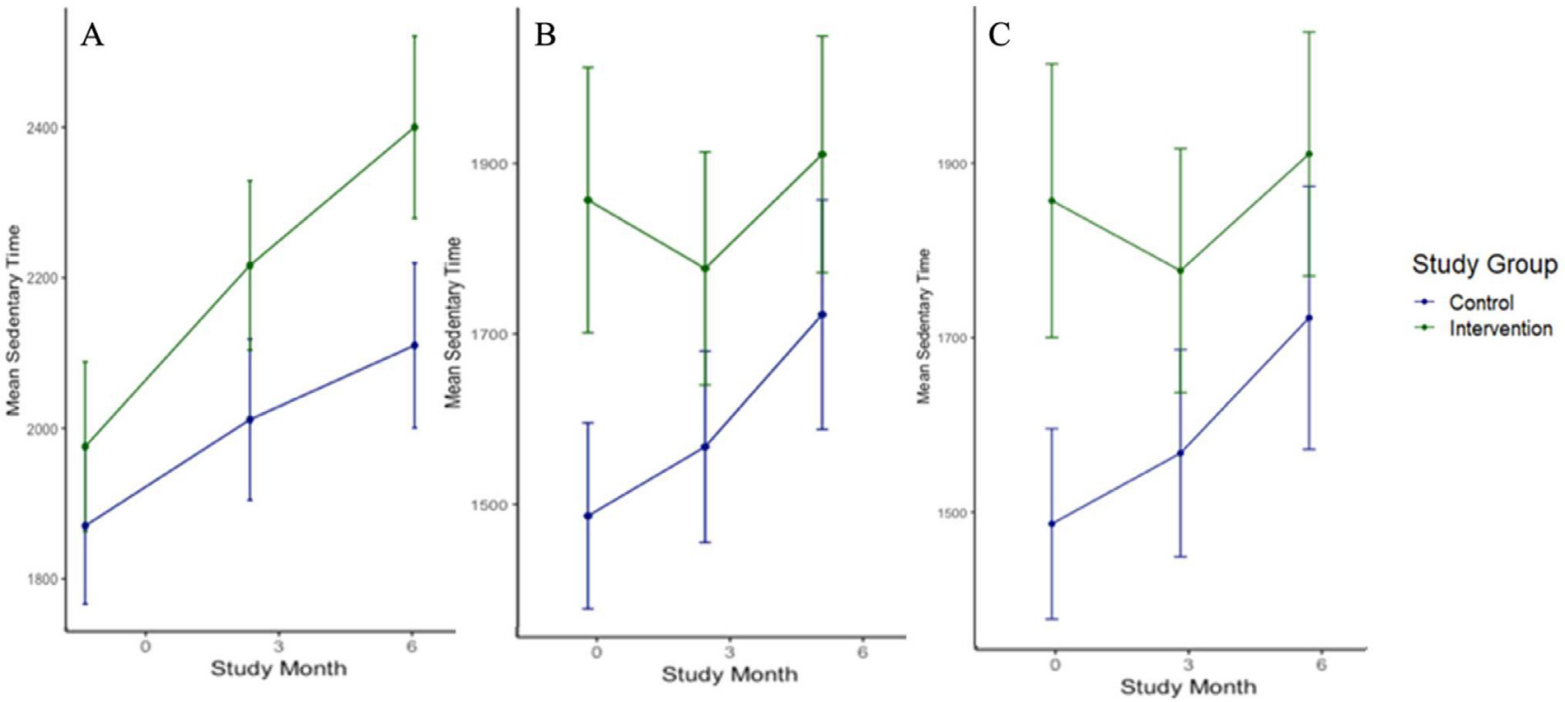
Longitudinal changes in sedentary time. (A) Changes in mean sedentary time (PA) among all participants. (B) Changes in mean sedentary time (PA) among adults. (C) Changes in mean sedentary time (PA) among all adult complete cases. Sedentary time units are provided as mins/wk. *Note:* In both groups, sedentary time increased during the trial (Panel A); however, the intervention interaction term was nonsignificant (*p*=0.49) (Table 4). 3mo, 3 months; 6mo, 6 months; mins/wk, minutes per week; PA, physical activity.

In both groups, sedentary time increased during the trial (Figure 4A); however, the intervention interaction term was nonsignificant (*p*=0.49) (Table 4). Although age was a significant covariate for analyses of sedentary time (*p*<0.01 among all participants and *p*=0.016 among adults), even after age stratification, the intervention interaction term remained nonsignificant throughout the trial among each of the groups (Figure 4A and B). Complete case analyses were similar (Figure 4C).

**Table 4:** Longitudinal Changes in Sedentary Time

Dependent variable	Coefficient	95% CI	*p*-value
All participants			
Intervention	13.02	−89.02, 697.32	0.94
Study Month 3	180.16	−174.11, 365.44	0.13
Study Month 6	310.11	−40.24, 520.99	0.01*
Age	−25.26	−22.13, −2.41	**<0.01^⁎⁎^**
Intervention Х study Month 3	62.63	−566.69, 206.85	0.71
Intervention Х study Month 6	119.50	−567.47, 217.38	0.49
Adult participants only			
Intervention	304.15	−89.02, 697.32	0.13
Study Month 3	95.67	−174.11, 365.44	0.49
Study Month 6	240.38	−40.24, 520.99	0.09
Age	−12.27	−22.13, −2.41	0.02*
Intervention Х study Month 3	−179.91	−566.69, 206.85	0.36
Intervention Х study Month 6	−175.04	−567.47, 217.38	0.38
Adults only, complete cases			
Intervention	317.57	−75.59, 710.73	0.11
Study Month 3	80.47	−192.00, 352.94	0.56
Study Month 6	215.37	−65.28, 496.02	0.13
Age	−10.69	−20.63, −0.75	0.04*
Intervention Х study Month 3	−550.79	−550.79–223.34	0.41
Intervention Х study Month 6	−583.53	−583.53, 202.13	0.34

*Note*: Boldface indicates statistical significance (*p<0.05, **p<0.01, and ***p<0.001).

Summary statistics from linear mixed-effects models for sedentary time among the control versus the intervention group are presented.

### Anthropometric Measures

There were no statistically significant changes among or between the groups in adult BMI (Appendix Figure 5A, available online), SBP (Appendix Figure 5B, available online), or DBP (Appendix Figure 5C, available online) throughout the trial (Appendix Table 3, available online). Similarly, when assessed among all participants, there were no statistically significant changes among or between the groups in BMI (Appendix Figure 5D, available online), SBP (Appendix Figure 5E, available online), or DBP (Appendix Figure 5F, available online) throughout the trial (Appendix Table 3, available online).

## DISCUSSION

To our knowledge, this study is the first RCT to assess the effect of a specific nutritional intervention on PA outcomes and extends the literature for the potential role of avocados in beneficial lifestyle modification in Hispanic/Latino families. The primary outcome of this study suggests that higher avocado intake may lead to significant improvements in total PA levels. Moreover, there were no adverse outcomes reported by participants, and clinical measurements were not different between the groups (i.e., no significant increase in BMI nor SBP or DBP). The effect was not due to overall calorie consumption. As such, modifiable lifestyle interventions, such as the dietary intake of avocados, may have beneficial and important pleiotropic effects.

The long-term public health impact of increased avocado intake and improved PA levels remains unclear. Clinical effects of PA improvements accrue over a lifetime.^37^ PA changes may take longer than 6 months to manifest clinical outcomes such as improvements in BMI, waist-to-height ratio, and blood pressure.^38^ Future studies may assess the dietary intervention and PA outcomes over an extended period, specifically powered for cardiometabolic clinical outcomes. The impact among the population included in this trial may be particularly beneficial, given that Hispanic/Latino individuals report the lowest levels of PA in the U.S.,^11^ report the highest rates of sedentary time,^10^ and are generally at higher risk of cardiometabolic disease.^14^^,^^15^ In light of current evidence that acculturation to a Western lifestyle can result in adverse health outcomes among Hispanic/Latino populations,^39^^,^^40^ it is also imperative to identify beneficial changes that can be easily incorporated into culturally traditional diets. Previous cross-sectional studies report a positive association between fruit and vegetable consumption and PA levels, congruent with the results of this longitudinal trial.^17^^,^^41^ Although a causal relationship is still unclear, and confounding factors such as wider health-conscious patterns may be contributory, this investigation does suggest that robustly increasing avocado intake, specifically among Latino adults, may result in improved PA levels.

There are several hypotheses that may account for these findings. It is possible that increased satiety^42^ and energy levels are associated with higher avocado intake, given the nutritionally dense content, allowing individuals to participate in more PA. Indeed, as previously reported in this cohort, the higher allotment of avocados was actually associated with a significantly reduced overall energy intake (–29% in the intervention group compared with just –3% in the control group).^19^ However, this study found that the improved PA levels in the intervention group were not due to changes in overall calorie consumption. Specific nutrients and pleiotropic effects of avocado intake may support higher PA levels.

One hypothesis is that the unsaponifiable components of avocados may improve pain regulation, given that studies have demonstrated that avocado intake is associated with statistically significant pain improvements in patients with osteoarthritis.^43^, ^44^, ^45^ As such, PA may naturally increase as participants feel better.^46^ In this investigation, the significant increase in PA measures among adults, although not significant among adolescents or children, may further support this hypothesis given that adults are more likely to have osteoarthritis or injuries. However, to our knowledge, no study has shown an increase in PA relevant to avocado intake. Further trials with avocado supplementation in specific subgroups and for longer periods may better elucidate the effect of this intervention and its relationship with pain regulation and PA.

### Limitations

This study has some limitations. For instance, the primary outcome of between-group differences in total PA was in part due to an unexpected decrease in PA among the control group. The allotment of avocados for the control group was based on intake survey data so that participants in the control group would not significantly change their overall avocado intake, and adherence data confirmed that avocado intake did not decrease in the control group. As such, the significant decrease in total PA in the control group is not explained by the study intervention. In fact, adherence data suggest that significant between-group differences may be attenuated given that the intervention group achieved slightly below the goal intake, and the control group reported slightly higher than the goal intake. Another limitation of this investigation is the finding that the largest degree of change in adult PA occurred among the specific category type of occupational PA, which is expected to be less volitional than recreational or transportation-related PA measures. Related to this concern is the limitation in this study's design of collecting PA data through participant-reported questionnaires. This may result in recall and response bias.^47^ Because it is reasonable to assume that both groups would be affected, between-group differences were assessed to decrease this effect. Among the available questionnaire methods, GPAQ used in this trial is well validated, including in Latino populations.^32^^,^^33^ Future studies may improve data quality using accelerometers and other types of wearable technology to measure PA time and effort levels more accurately.

Another important limitation of this study is the inability to blind participants and community healthcare workers to the intervention, which risks information bias and introduces the potential for differential treatment of the control group unrelated to the actual consumption of the avocado intervention. Although participants in the control group were not made aware of how many avocados were aliquoted to the intervention group, they still were aware that they were not to change their avocado consumption from before the trial enrollment. The difference in attrition rates between the intervention and control groups may have been affected by the insight that they were assigned to the group receiving a lower aliquot number, given that dropout only occurred in the control group. To better assess this observation, it was confirmed that there were no significant differences at baseline between those who dropped out and those who remained in the trial. To best mitigate the risk of not blinding, both groups were treated as equally as possible other than the direct allotment number of avocados. Indeed, both groups received standardized health education counseling by the community health workers, and both groups attended clinic visits for questionnaires and anthropometric measures.

This investigation has several strengths, including the RCT design clustered per family, with statistical methods to assess longitudinal changes in primary and secondary outcomes. Compared with most nutritional intervention studies, the trial period of 6 months was relatively long. This study also uniquely incorporated data on baseline avocado intake to design a control study arm assignment as the ref rather than comparing with no avocado intake, which could have resulted in a confounding effect, and allowed the trial to be more culturally applicable for the enrolled Hispanic/Latino population.^48^ However, future studies are needed to assess findings in multiethnic populations. Finally, considering an implementation context, behaviorally based lifestyle interventions—for example, an increase in dietary avocado intake—are likely synergistic with standard exercise programs and more cost effective than a structured exercise program in improving PA levels.^49^ Further trials are needed to compare intervention effects.

## CONCLUSIONS

Compared with a lower amount, a higher allocation of avocados in Hispanic/Latino families resulted in significant increases in total PA. Such an effect may lead to beneficial outcomes at the population level, especially if accrued over several years. As such, when combined with standardized nutrition education, we advocate that higher avocado intake be incorporated into a healthy diet for Hispanic/Latino families, and we do not expect adverse outcomes. The findings suggest that this specific nutritional lifestyle intervention may have beneficial pleiotropic effects.

## CRediT authorship contribution statement

**Tara Shrout Allen:** Conceptualization, Methodology, Validation, Formal analysis, Data curation, Writing – original draft, Writing – review & editing, Visualization, Project administration. **Aubrey L. Doede:** Formal analysis, Data curation, Writing – original draft, Writing – review & editing, Visualization. **Colin M.B. King:** Data curation, Writing – original draft. **Lorena S. Pacheco:** Investigation, Resources, Writing – review & editing. **Gregory A. Talavera:** Validation, Investigation, Writing – review & editing, Supervision. **Julie O. Denenberg:** Resources, Data curation, Project administration. **Amelia S. Eastman:** Writing – review & editing. **Michael H. Criqui:** Writing – review & editing, Supervision. **Matthew A. Allison:** Conceptualization, Methodology, Resources, Writing – original draft, Writing – review & editing, Supervision, Project administration, Funding acquisition.

## Appendix Supplementary materials

Image, application 1

Image, application 2

## Footnotes

Supplementary material associated with this article can be found in the online version at doi:10.1016/j.focus.2023.100145.
