# Supplementary material for: Nutritional Avocado Intervention Improves Physical Activity Measures in Hispanic/Latino Families: A Cluster RCT
Source: AJPM Focus. 2023 Sep 20;2(4):100145. doi: 10.1016/j.focus.2023.100145 (PMC10628653; doi:10.1016/j.focus.2023.100145)
Supplement: Supplementary file 1 [file mmc1.docx]

**Fig S1.** Avocado Daily Diary used as an Intervention Adherence Tool

**Week Beginning Date: __________________________________________**

Please provide us with some information about your family’s avocado intake this week, by completing the table below. We are primarily interested in whether or not the foods you and your family prepared today contained avocado, how much avocado was consumed, and how each food was prepared. Additionally, please tell us if you used any of the recipes on the Avocado Recipe Booklet, by writing the recipe and/or page number in area marked “Recipe*s”*. Also, please share any other notes and/or comments you would like to share.

**Instructions:** To be completed by the member of the family most responsible for preparing and/or serving food for the family.

**Avocado Intake:** Specify if you had avocado that day as a family by circling “Yes” or “No”.

**Amount of *avocado* that you ate or drank *as a family*:** Specify whether it was <¼ (less than ¼), ¼, ½, ¾, 1, 2, or 3 whole avocados, or don’t know. For 2 ½ avocado, circle “2” and “½”.

**Avocado preparation:** Circle if it was “Cooked”, “Raw”, or “Both”.

| **Time/Meal** | **Did your family consume avocado today?** | **How much avocado**  **did your family consume today?** | | **How was it prepared?** |
| --- | --- | --- | --- | --- |
| **Monday**  Date: | Yes No | < ¼ ¼ ½ ¾ 1 2 3 don’t know  Notes:____________________________________ | | Cooked Raw Both  Recipes: _________________ |
| **Tuesday**  Date: | Yes No | < ¼ ¼ ½ ¾ 1 2 3 don’t know  Notes: ____________________________________ | | Cooked Raw Both  Recipes: _________________ |
| **Wednesday**  Date: | Yes No | < ¼ ¼ ½ ¾ 1 2 3 don’t know  Notes: ___________________________________ | | Cooked Raw Both  Recipes: _________________ |
| **Thursday**  Date: | Yes No | < ¼ ¼ ½ ¾ 1 2 3 don’t know  Notes: ____________________________________ | | Cooked Raw Both  Recipes: _________________ |
| **Friday**  Date: | Yes No | < ¼ ¼ ½ ¾ 1 2 3 don’t know  Notes: ___________________________________ | | Cooked Raw Both  Recipes: _________________ |
| **Saturday**  Date: | Yes No | < ¼ ¼ ½ ¾ 1 2 3 don’t know  Notes: ____________________________________ | | Cooked Raw Both  Recipes: _________________ |
| **Sunday**  Date: | Yes No | < ¼ ¼ ½ ¾ 1 2 3 don’t know  Notes: ____________________________________ | | Cooked Raw Both  Recipes: _________________ |
| **Weekly Summary** | How many days this week did you eat avocado?  **_______________________** | | How much avocados does your family have left this week?  ___________________________ | |
| **Comments:** ___________________________________________________________________________________________________________  _____________________________________________________________________________________________________________________ | | | | |

**Fig S2** . Individual Avocado Intake per Week per Study Group


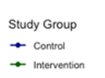

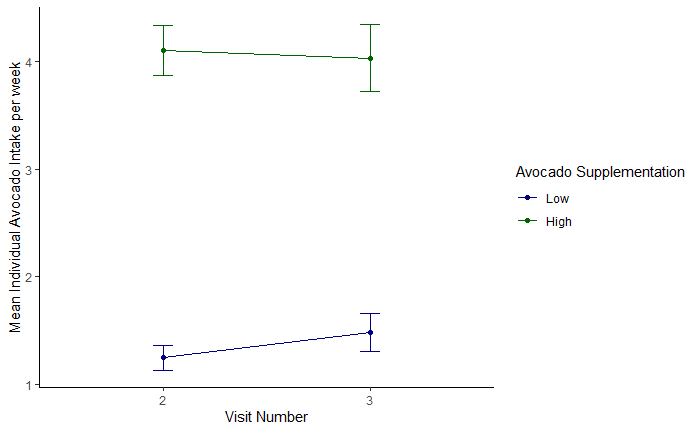


Individuals in the intervention group had an average intake of 4.10 and 4.03 avocados/week at the second (3-month) and third (6-month) clinic visits, respectively, which was slightly below the goal of approximately 4.24 avocados/week/individual, accounting for mean family size of the intervention group. Individuals in the control group had an average intake of 1.25 and 1.48 avocados/week at the 3- and 6-month clinic visits, respectively, which was on average slightly above the goal of approximately 0.94 avocados/week/individual, accounting for mean family size of the control group. Visit Number 2 corresponds to the study clinic visit at 3 months (trial mid-point); Visit Number 3, study clinic visit at 6 months (trial endpoint).

**Fig S3**. Longitudinal Changes in Physical Activity Measures among All Participants

A

B

C

**
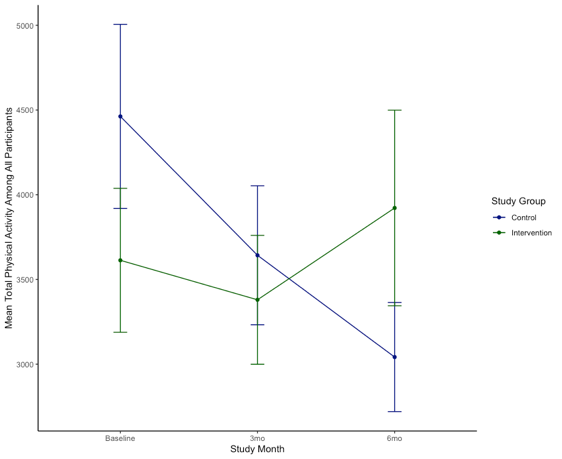

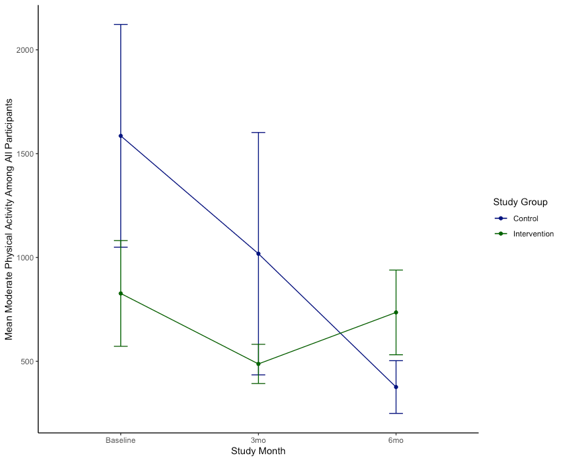

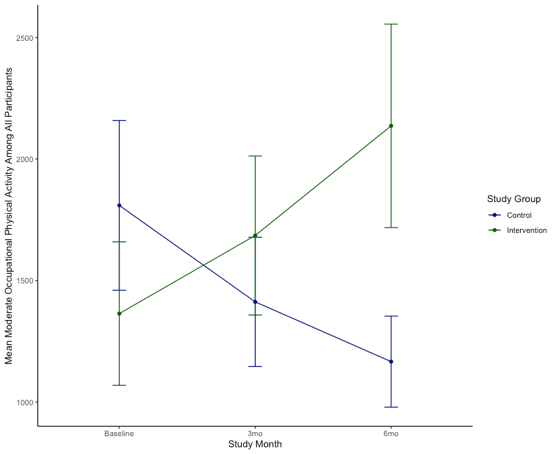
**

Among all participants, linear mixed effects models demonstrated that total physical activity (PA) (S3A) significantly improved by the trial endpoint in the intervention versus control group. There was no significant change between groups in moderate PA (S3B) or moderate occupational activity (S3C) when assessed among all participants. PA units as MET-mins/week, metabolic equivalent minutes per week. Study month, 3mo indicates the 3-month mid-point visit, and 6mo indicates the 6-month trial endpoint visit.

**Fig S4**. Longitudinal Changes in Physical Activity Measures among Children and Adolescents

C

B

A


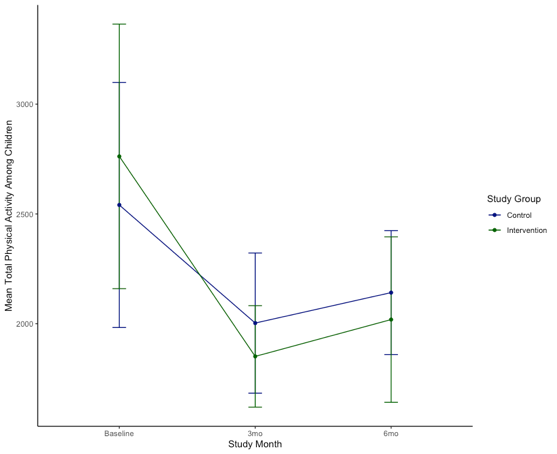

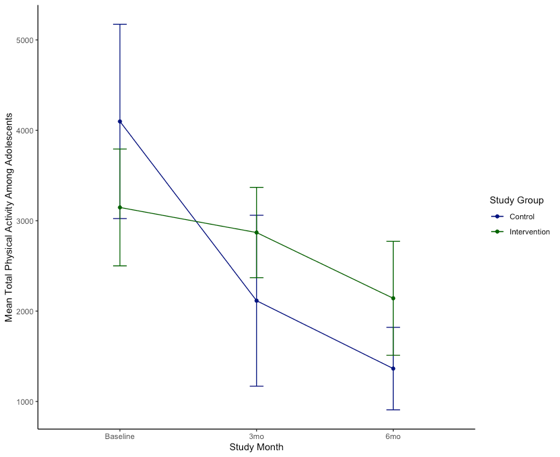

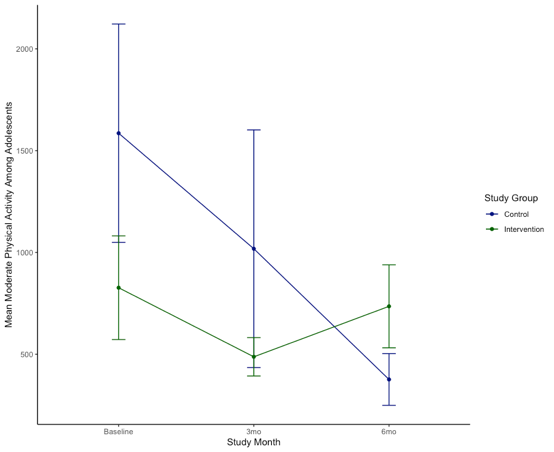


Linear mixed effects models demonstrated that total physical activity (PA) among children (S4A) and adolescents (S4B) did not significantly differ between the intervention versus control group by the mid-point nor by the endpoint of the trial. Similarly, the PA sub-category of moderate PA in adolescents did not significantly differ between groups (S4C). PA units as MET-mins/week, metabolic equivalent minutes per week. Study month, 3mo indicates the 3-month mid-point visit, and 6mo indicates the 6-month trial endpoint visit.

**Fig S5A-S5F:** Longitudinal Changes in Anthropometric Measures among All Participants and Stratified to Adults

**
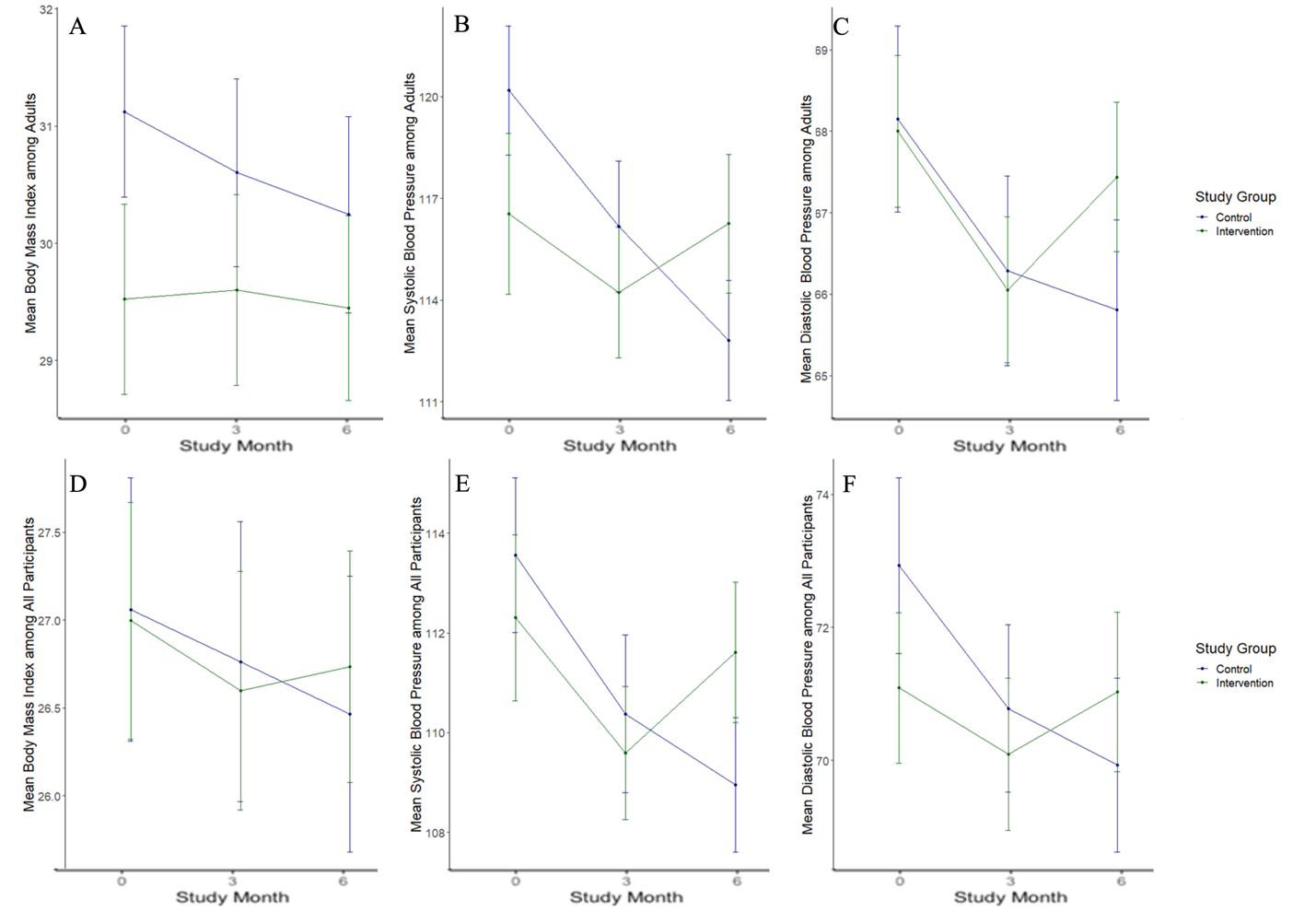
**

Linear mixed effects models demonstrated that there were no statistically significant changes in longitudinal anthropometric measures among adults (5A-5C) or among all participants (5D-5F), including that of body mass index (BMI) (5A; 5D), systolic blood pressure (SBP) (5B; 5E), or diastolic blood pressure (DBP) (5C; 5F), respectively. Units for BMI are kg/m^2^  and units for SBP and SBP are mmHg. Study month, 3mo indicates the 3-month mid-point visit, and 6mo indicates the 6-month trial endpoint visit.

**Table S1.** Baseline Characteristics of Families that Completed versus Did Not Complete the Trial

| **Baseline Characteristic** | **Families that completed the study** | | **Families that dropped out** | | ***p* value^1^** |
| --- | --- | --- | --- | --- | --- |
| **Families** | **N = 66** | | **N = 6** | |  |
|  | **N** | **%** | **N** | **%** |  |
| **Family income <$30k, USD/year** | 32 | 44.4 | 2 | 2.8 | 0.48 |
|  | **Mean** | **SD** | **Mean** | **SD** |  |
| **Mean family size** | 3 | 0.5 | 3 | 0.5 | 0.58 |
| **Adult body mass index**  **Systolic BP, mmHg**  Adults  Adolescents  Children  **Diastolic BP, mmHg**  Adults  Adolescents  Children  **Total PA, MET-mins/wk** | 30.0  117  110  101  71.5  65.3  59.9 | 6.4  16.7  8.1  7.3  10.5  8.4  7.6 | 32.9  125  110  97.1  74.3  62.5  54.7 | 6.6  24.0  5.8  9.1  9.5  5.5  9.9 | 0.80  0.87  0.89  0.36  0.29  0.41  0.22 |
| Adults | 4378 | 5568 | 7925 | 10,014 | 0.20 |
| Adolescents | 2577 | 2739 | 7470 | 3052 | **0.04*** |
| Children | 2542 | 3051 | 3866 | 4336 | 0.46 |
| **Sedentary time, mins/wk** |  |  |  |  |  |
| Adults | 1668 | 1156 | 1605 | 803 | 0.79 |
| Adolescents | 2213 | 1179 | 2572 | 1556 | 0.68 |
| Children | 2293 | 1094 | 3180 | 1134 | 0.09 |
|  |  |  |  |  |  |
| **Head of household** | **From families that completed the study** | | **From families that dropped out** | |  |
|  | **Mean** | **SD** | **Mean** | **SD** |  |
| **Years lived in the United States** | 17.4 | 12.6 | 14.2 | 14.4 | 0.56 |
|  | **N** | **%** | **N** | **%** |  |
| **Female** | 65 | 90.3 | 6 | 8.3 | 0.76 |
| **Country of birth, Mexico** | 56 | 77.8 | 4 | 5.6 | 0.25 |
| **Heritage, Mexican** | 64 | 88.9 | 6 | 8.3 | 0.67 |
| **Marital status** |  |  |  |  | 0.76 |
| Married or cohabitation | 44 | 61.1 | 4 | 5.6 |  |
| Separated, divorced, or widowed | 8 | 11.1 | 1 | 1.4 |  |
| Single | 14 | 19.4 | 1 | 1.4 |  |
| **Highest level of education** |  |  |  |  | 0.85 |
| High school | 17 | 23.6 | 2 | 2.8 |  |
| Trade school or Associate’s degree | 19 | 26.4 | 1 | 1.4 |  |
| Bachelor’s degree or above | 15 | 20.8 | 1 | 1.4 |  |
| No diploma | 7 | 9.7 | 0 | 0 |  |
| Other | 8 | 11.1 | 2 | 2.8 |  |
| **Country where highest level of**  **education was completed** |  |  |  |  | 0.51 |
| United States or other | 24 | 33.3 | 3 | 4.2 |  |
| Mexico | 42 | 58.3 | 3 | 4.2 |  |
| **Employment status** |  |  |  |  | 0.93 |
| Employed for wages | 16 | 22.5 | 3 | 4.2 |  |
| Self-employed | 6 | 8.5 | 1 | 1.4 |  |
| Homemaker | 30 | 42.3 | 2 | 2.8 |  |
| Other | 13 | 18.2 | 0 | 0 |  |

^1^ From unpaired t-test or chi-square where appropriate. Statistical significance designated with asterisks as follows: *p<0.05, **p<0.01, ***p<0.001. BP, blood pressure. MET, metabolic equivalents. Mins, minutes. Wk, week. PA, physical activity. SD, standard deviation. USD, United States Dollars.

**Table S2. Longitudinal Changes in Physical Activity Measures among All Participants**

| **Dependent Variable** | **Coefficient** | **95% CI** | **p** |
| --- | --- | --- | --- |
| **Total Physical Activity (PA)** |  |  |  |
| Intervention | -836 | -2223, 4799 | 0.23 |
| Study month 3 | -829 | -1853, 196 | 0.11 |
| Study month 6 | -1447 | -2485, -408 | **0.006**** |
| Intervention * Study month 3 | 418 | -998, 1834 | 0.56 |
| Intervention * Study month 6 | 1634 | 204, 3065 | **0.03*** |
| **Moderate PA** |  |  |  |
| Intervention | -218 | -823, 1445 | 0.47 |
| Study month 3 | 227 | -758, 304 | 0.40 |
| Study month 6 | -357 | -903, 190 | 0.20 |
| Intervention * Study month 3 | 215 | -528, 957 | 0.57 |
| Intervention * Study month 6 | 897 | 143, 1651 | **0.02*** |
| **Moderate Occupational PA** |  |  |  |
| Intervention | -255 | -832, 322 | 0.38 |
| Study month 3 | -221 | -720, 278 | 0.39 |
| Study month 6 | -434 | -948, 80 | 0.10 |
| Intervention * Study month 3 | 379 | -324, 1081 | 0.29 |
| Intervention * Study month 6 | 874 | 161, 1587 | **0.02*** |

Linear mixed effects models summary statistics for physical activity measures among the intervention versus control group among all participants. Statistical significance designated with asterisks as follows: *p<0.05, and **p<0.01, ***p<0.001. CI, confidence interval. PA, physical activity. Study month, 3mo indicates the 3-month mid-point visit, and 6mo indicates the 6-month trial endpoint visit.

**Table S3. Sensitivity Analysis: Longitudinal Changes in Total Physical Activity Accounting for Total Energy Intake**

| **Dependent Variable** | **Coefficient** | **95% CI** | **p** |
| --- | --- | --- | --- |
| **Total PA among All participants** |  |  |  |
| Intervention | -967 | -2363, 429 | 0.17 |
| Study month 3 | -803 | -1846, 239 | 0.13 |
| Study month 6 | -1459 | -2515, -403 | **0.007**** |
| Intervention * Study month 3 | 655 | -790, 2101 | 0.37 |
| Intervention * Study month 6 | 1981 | 510, 3452 | **0.009**** |
| **Total PA among Adults** |  |  |  |
| Intervention | -1643 | -3768, 483 | 0.13 |
| Study month 3 | -867 | -2411, 676 | 0.27 |
| Study month 6 | -1737 | -3319, -155 | **0.03*** |
| Intervention * Study month 3 | 1037 | -1181, 3256 | 0.36 |
| Intervention * Study month 6 | 3246 | 971, 5520 | **0.005**** |

Linear mixed effects models summary statistics for physical activity measures among the control versus intervention group. Statistical significance designated with asterisks as follows: *p<0.05, and **p<0.01, ***p<0.001. CI, confidence interval. PA, physical activity.

**Table S4**. Anthropometric Measures by Study Group throughout the Trial

|  | **Control Group** | | | **Intervention Group** | | |
| --- | --- | --- | --- | --- | --- | --- |
|  | **(37 families; 120 participants)** | | | **(35 families; 115 participants)** | | |
|  | **N** | **Mean** | **Std. Dev.** | **N** | **Mean** | **Std. Dev** |
| **BMI – All participants** |  |  |  |  |  |  |
| Baseline | 116 | 26.9 | 7.95 | 110 | 26.9 | 7.07 |
| Study month 3 | 101 | 26.7 | 7.85 | 114 | 26.6 | 7.09 |
| Study month 6 | 92 | 26.5 | 7.53 | 114 | 26.7 | 6.89 |
| **BMI – Adults only** |  |  |  |  |  |  |
| Baseline | 73 | 30.9 | 6.40 | 67 | 29.6 | 6.55 |
| Study month 3 | 65 | 30.6 | 6.39 | 66 | 29.5 | 6.46 |
| Study month 6 | 58 | 30.2 | 6.38 | 66 | 29.4 | 6.25 |
| **SBP – All participants** |  |  |  |  |  |  |
| Baseline | 116 | 113 | 16.5 | 110 | 112 | 17.2 |
| Study month 3 | 101 | 110 | 15.5 | 114 | 110 | 14.5 |
| Study month 6 | 92 | 109 | 12.9 | 114 | 112 | 14.6 |
| **SBP – Adults only** |  |  |  |  |  |  |
| Baseline | 73 | 120 | 16.3 | 67 | 117 | 19.2 |
| Study month 3 | 65 | 116 | 15.3 | 66 | 115 | 16.0 |
| Study month 6 | 58 | 113 | 13.5 | 66 | 116 | 16.3 |
| **DBP – All participants** |  |  |  |  |  |  |
| Baseline | 116 | 67.9 | 12.1 | 110 | 68.1 | 9.55 |
| Study month 3 | 101 | 66.3 | 11.5 | 114 | 66.3 | 9.79 |
| Study month 6 | 92 | 65.8 | 10.6 | 114 | 67.4 | 9.52 |
| **DBP – Adults only** |  |  |  |  |  |  |
| Baseline | 73 | 72.7 | 11.3 | 67 | 71.2 | 9.18 |
| Study month 3 | 65 | 70.8 | 10.0 | 66 | 70.4 | 9.64 |
| Study month 6 | 58 | 69.9 | 9.95 | 66 | 71.0 | 9.50 |

BMI, body mass index. DBP, diastolic blood pressure. SBP, systolic blood pressure.
